# Supplementary material for: Comparison of the In Vivo Efficacy of Cuban (Raydel®) and Chinese (BOC Science) Policosanol in Alleviating Dyslipidemia and Inflammation via Safeguarding Major Organs and Reproductive Health in Hyperlipidemic Zebrafish: A Twelve-Week Consumption Study
Source: Pharmaceuticals (Basel). 2024 Aug 22;17(8):1103. doi: 10.3390/ph17081103 (PMC11357553; doi:10.3390/ph17081103)
Supplement: Supplementary file 1 [file pharmaceuticals-17-01103-s001.zip › pharmaceuticals-3129130-supplementary.pdf]

Supplementary Material

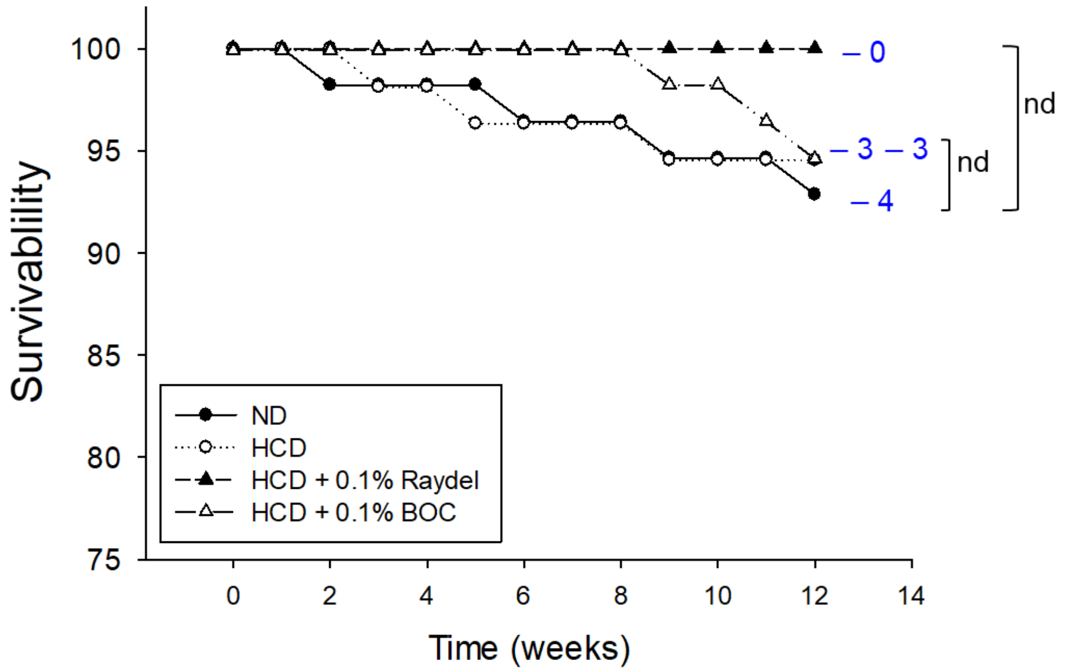

Supplementary Figure S1. Zebrafish survivability across the different groups during 12 weeks. ND denotes normal diet, HCD denotes the high cholesterol diet (i.e., ND containing 4% cholesterol), while HCD + Raydel® or BOC represent the HCD+0.1% Raydel®-policosanol or BOC sciences-policosanol, respectively. The "nd" non-significant difference between the groups.

Supplementary Material S1:

1. List of the used chemicals: N-ε-carboxylmethyllysine (CAS-No 941689-36-7, Cat#14580-5g), dihydroethidium (DHE, 104821-25-2, Cat #37291), and acridine orange (AO, 65-61-2, Cat#A9231), oil red O (Cat#O0625), and 2-phenoxyethanol (Sigma P1126; St. Louis, MO, USA), paraoxon-ethyl (Cat. No. 36186) and 5-bromo-4-chloro-3-indolyl β D-galactopyranoside (X-gal, Cat#B54252) were procured from Sigma–Aldrich (St. Louis, MO, USA). All other chemicals and reagents else otherwise stated were of analytical grade and used as supplied.

2. Analysis of Plasma

Blood samples (2 μL) were collected from the hearts of adult zebrafish and combined with 3 μL of phosphate-buffered saline (PBS)-ethylenediaminetetraacetic acid (EDTA, 1 mM). The blood of each zebrafish (2 μL) from the specified group was pooled (n=8~10) in a single tube containing (EDTA, 1 mM). The pooled samples obtained from the different groups were centrifuged at 3,000 rpm for 10 min

to obtain plasma. The plasma from the different groups wase collected in separate tubes and preserved at low temperature (4°C) for further analysis.

Plasma total cholesterol (TC) and triglycerides (TG) were measured using commercial assay kits (cholesterol, T-CHO, and TGs, Cleantech TS-S; Wako Pure Chemical, Osaka, Japan) according to the manufacturer's protocols. For TC analysis, 5 μL of plasma was mixed with 200 μL of the reaction mixture provided in the assay kit and incubated at 37°C for 10 minutes, resulting in a dark pink product measured at 450 nm using a microplate reader (Bio-Rad, Hercules, CA, USA).

Similarly, for TG analysis, 5 μL of plasma was combined with 200 μL of the TG-specific reaction mixture from the assay kit and incubated at 37°C for 10 min. The resulting purple product was measured at an absorbance of 490 nm. For HDL-C analysis, plasma was mixed with an equal volume of separation solution from the assay kit and centrifuged at 3,000 rpm for 10 min. The supernatant (20 μL) was mixed with 200 μL of the TC reagent reaction mixture and incubated at 37°C for 10 min. The dark pink color intensity, corresponding to HDL-C, was measured at an absorbance of 490 nm.

To quantify aspartate transaminase (AST) and alanine transaminase (ALT) levels, a commercial diagnostic kit (Asan Pharmaceutical, Hwasung, Republic of Korea) was used, following the manufacturer's instructions. Briefly, 5 μL of plasma was combined with 250 μL of AST or ALT-specific solution from the kit. The mixture was incubated at 37°C for 30 min for AST and 60 min for ALT. Following incubation, 250 μL of the respective coloring reagent was added, and the mixture was incubated at room temperature for 20 min. The reaction was then stopped by adding 250 μL of 0.4 N NaOH, and AST and ALT levels were quantified by measuring absorbance at 490 nm.
